# A Review of Sodium-Glucose Cotransporter 2 Inhibitor’s Clinical Efficacy in Heart Failure With Preserved Ejection Fraction

**DOI:** 10.7759/cureus.57380

**Published:** 2024-04-01

**Authors:** Jacob Epperson, Zoraize Moeez Athar, Mahnoor Arshad, Edward Chen

**Affiliations:** 1 Internal Medicine, BronxCare Health System, New York, USA

**Keywords:** sodium-glucose cotransporter (sglt) efficacy, sodium-glucose cotransporter-2 (sglt2) inhibitors, sglt2 inhibitors and heart failure, hfpef, heart failure with preserved ejection fraction (hfpef)

## Abstract

Heart failure (HF) poses a significant healthcare burden, with distinct subtypes based on ventricular function. HF with preserved ejection fraction (HFpEF) presents unique epidemiological and mechanistic features compared to HF with reduced ejection fraction (HFrEF). The pathophysiology of HFpEF is complex and involves multiple factors. Current pharmacological therapies for HFpEF remain suboptimal, with inconsistent mortality outcomes observed despite improvements in symptoms and quality of life. Sodium-glucose cotransporter 2 (SGLT2) inhibitors have emerged as promising agents in HF management and hospitalizations, particularly in HFpEF patients. The cardioprotective mechanisms of SGLT2 inhibitors are multifactorial. In this article, we performed a comprehensive review of SGLT2 inhibitor use in HFpEF and discussed the implications in the management of HF.

## Introduction and background

Heart failure (HF)

Heart failure (HF) encompasses two distinct subtypes that are contingent upon the function of the right ventricle (RV) and left ventricle (LV). Heart failure with preserved ejection fraction (HFpEF), also known as diastolic failure, presents epidemiological and mechanistic disparities compared to HF with reduced ejection fraction (HFrEF). HFpEF, as defined by the American Heart Association, is characterized by a left ventricular ejection fraction (LVEF) that is greater than or equal to 50% and constitutes nearly half of all HF cases [1]. It exhibits a mortality rate comparable to HFrEF, which might stem from the disease's progression, with many patients exhibiting diminished myocardial compliance alongside comorbidities such as hypertension, concentric hypertrophy, and decreased diastolic function [2,3,4].

Ventricular dysfunction in HFpEF encompasses prolonged active LV relaxation, exacerbated during exertion, and a loss of the intraventricular pressure gradient known as "cardiac suction." Consequently, left atrial (LA) hypertension becomes crucial to drive left ventricular filling pressure (LVFP), leading to cardiac remodeling and potentially pulmonary hypertension, observed in approximately 30% of HFpEF patients. Elevated pulmonary vascular pressures often result in right heart dysfunction. This predisposes patients to adverse outcomes via venous congestion [5,6].

Despite these findings, the pathophysiology of HFpEF remains largely elusive. While coronary microvascular dysfunction is a significant factor in disease development, recent data suggest that extracardiac pathologies such as obesity and diabetes mellitus (DM) may play a larger role than previously thought [5]. Factors such as epicardial fat expansion and LV fibrosis, triggered by obesity and DM, can induce hemodynamic instability, which leads to increased LVFP. Cadaveric studies in HFpEF patients have revealed a correlation between reduced coronary microvasculature density and myocardial fibrosis magnitude [5]. Additionally, vascular abnormalities such as peripheral vascular disease and endothelial dysfunction, along with decreased nitric oxide levels seen in HFpEF, contribute to greater afterload and increased end-systolic volume (ESV). Although rare, infiltrative cardiomyopathies such as amyloidosis are estimated to affect roughly 15-20% of HFpEF patients [6,7].

Notably, renal function serves as a significant independent predictor of mortality in HFpEF, with 50-60% of HFpEF patients having chronic kidney disease (CKD) [8]. Within the context of CKD, hypertension emerges as the primary attributable risk factor for diastolic dysfunction, prompting therapeutic strategies to be predominantly aimed at managing hypertension. Despite trial evidence displaying inconsistent and largely neutral outcomes regarding mortality improvement, various studies have indicated that pharmacological therapy could enhance exercise tolerance and quality of life [3]. While treatments such as beta-blockers (BB), angiotensin-converting enzyme (ACE) inhibitors, angiotensin receptor blockers (ARB), diuretics, mineralocorticoid antagonists (MRA), vasodilators, and angiotensin receptor blocker-neprilysin inhibitors (ARNI) have shown efficacy in HFrEF, their effectiveness in HFpEF is often evaluated based on their impact on hospitalization, functional status, symptoms, and quality of life because of the increased frequency of presentation in patients of older age and increased comorbidities [9]. However, these therapies have not consistently demonstrated efficacy in reducing morbidity or mortality in HFpEF [7,8].

## Review

HF management trials with SLGT2 inhibitors

HF management has seen the emergence of sodium-glucose cotransporter 2 inhibitors (SGLT2) as a strong contender among identified pharmacotherapies. Traditionally used as antidiabetic agents, SGLT2 inhibitors have demonstrated efficacy in slowing kidney disease progression and reducing the risk of end-stage renal disease (ESRD) in both type 2 DM (T2DM) and HFrEF patients, irrespective of diabetes status [8,9]. In 2021, dapagliflozin, an SGLT2 medication, received FDA approval for reducing CKD progression and mortality. In the recent Empagliflozin Outcome Trial in Patients with Chronic Heart Failure with Preserved Ejection Fraction (EMPEROR-Preserved) trial, approximately two years of SGLT2 treatment significantly slowed renal function decline and reduced HF-related hospitalizations in HFpEF patients [9,10]. However, its impact on mortality risk associated with ESRD in HFpEF patients has yet to be conclusively demonstrated [3,4,5,10]. Various studies have been performed to elucidate whether the implementation of SGLT2 inhibitors shows an improvement in mortality and morbidity avoidance in patients with HFpEF.

In the Dapagliflozin and Prevention of Adverse Outcomes in Heart Failure (DAPA-HF) trial, participants assigned to receive dapagliflozin exhibited a decreased risk of death from cardiovascular causes compared to those assigned to a placebo [11]. A meta-analysis combining this trial with others revealed no statistically significant heterogeneity of treatment effect concerning this outcome [11,12]. Further details are described in Table 1.

Regarding the primary outcome, which encompasses a composite of major adverse renal outcomes (marked and sustained declines in estimated glomerular filtration rate (eGFR) or the need for renal-replacement therapy), the EMPEROR-Preserved trial presented a significantly lower incidence compared to the Empagliflozin Outcome Trial in Patients with Chronic Heart Failure and a Reduced Ejection Fraction (EMPEROR-Reduced) trial [10]. These data indicate that empagliflozin may have a lesser kidney-protective effect in HF patients with preserved ejection fraction compared to those with reduced ejection fraction. Despite this discrepancy, both patient populations experienced a similar benefit concerning the risk of hospitalization for HF, which suggests that renal protection might not be the primary mechanism through which empagliflozin mitigates hospitalizations in HF [4,11].

The Canagliflozin Cardiovascular Assessment Study (CANVAS Program) and CANVAS-Renal (CANVAS-R) encompassed a cohort of 10,142 patients, with approximately two-thirds of them having cardiovascular disease [4]. The primary endpoint, major adverse cardiovascular events, exhibited a significant reduction (hazard ratio (HR) of 0.86, 95% confidence interval of 0.75 to 0.97) across various subgroups delineated by baseline hemoglobin A1C levels, kidney function, and duration and intensity of treatment for type 2 diabetes mellitus (T2DM). Notably, among these endpoints, HF demonstrated the most substantial reduction (HR 0.67, 95% confidence interval (CI) 0.52 to 0.87) [3,4].

The Dapagliflozin Effect on Cardiovascular Events-Thrombolysis in Myocardial Infarction 58 (DECLARE-TIMI) trial enrolled 17,160 patients with existing or at risk for atherosclerotic cardiovascular disease [2,3,4]. Notably, this trial featured a minimal-risk population, which, among outcomes trials conducted, is the lowest to date [4].

Regarding the primary endpoints, dapagliflozin did not demonstrate a reduction in cardiovascular adverse events. However, it did result in a lower incidence of cardiovascular death or hospitalizations for HF (HR 0.83; 95% CI 0.73 to 0.95) [4]. Furthermore, a significant reduction in cardiovascular mortality, as well as death from any cause, was observed among patients at high risk, including those with HFrEF and a history of myocardial infarction [2,3,4].

The Effect of Sotagliflozin on Cardiovascular Events in Patients with Type 2 Diabetes Post Worsening Heart Failure (SOLOIST-WHF) trial, comprising 1,222 patients recently hospitalized for decompensated HF, observed a primary endpoint reduction in the sotagliflozin group, with a HR of 0.67 (95% CI, 0.52 to 0.85) [4]. This primary endpoint was defined as a composite of cardiovascular death, hospitalizations for HF, or urgent visits for HF [2,3].

Concurrently, the Effect of Sotagliflozin on Cardiovascular and Renal Events (SCORED) study enrolled 10,584 patients diagnosed with T2DM and chronic kidney disease, all at risk for arteriosclerotic cardiovascular disease. Mirroring the primary endpoint of the SOLOIST-WHF trial, the SCORED trial also reported a significant reduction (HR 0.74; 95% CI, 0.63 to 0.88). Moreover, notable decreases in the total number of myocardial infarctions and strokes were observed [3].

The Cardiac and Metabolic Effects of Dapagliflozin in Heart Failure With Preserved Ejection Fraction (CAMEO-DAPA) evaluation represents a prospective study with the primary endpoint of interest involving the assessment of alterations in pulmonary capillary wedge pressure (PCWP) from baseline to the seventh-month mark during exercise while on treatment with dapagliflozin. Dapagliflozin demonstrated lower PCWP at rest (estimated treatment difference (ETD), -3.5 mmHg (95% CI, -6.6 to -0.4); p=0.029) and during maximal exercise (ETD, -5.7 mmHg (95% CI, -10.8 to -0.7); p=0.027) [13]. Additionally, treatment with dapagliflozin resulted in reductions in body weight (ETD, -3.5 kg (95% CI, -5.9 to -1.1); p=0.006) and plasma volume (ETD, -285 mL (95% CI, -510 to -60); p=0.014) [13], with no significant effect observed on red blood cell volume [14]. While there were no differences in oxygen consumption at 20-W or peak exercise, dapagliflozin decreased arterial lactate levels at 20 W (-0.70 ± 0.77 versus 0.37 ± 1.29 mm; p=0.006) [13].

SGLT2 inhibitors have been shown to enhance cardiovascular outcomes in patients with T2DM, but the comparative effectiveness of individual SGLT2 remains uncertain. This review conducted a systematic search across various peer-reviewed sources to identify randomized controlled trials investigating canagliflozin, dapagliflozin, empagliflozin, or ertugliflozin in T2DM patients. Placebo or any other active treatment served as comparators, with the primary endpoint being all-cause mortality and secondary endpoints including cardiovascular mortality and worsening HF.

Empagliflozin and canagliflozin were found to improve all three endpoints when compared with placebo, while dapagliflozin specifically improved worsening HF [14]. When comparing different SGLT2, empagliflozin exhibited superiority in reducing all-cause and cardiovascular mortality. Additionally, empagliflozin, canagliflozin, and dapagliflozin showed similar efficacy in improving worsening HF, whereas ertugliflozin had no discernible effect on any endpoint. Consistent results were observed when mortality analyses were restricted to patients enrolled in cardiovascular outcome trials. Empagliflozin emerged as superior in improving survival, while prospective head-to-head comparisons are warranted to validate these findings [6,7,11,14].

Table 1 summarizes the literature mentioned in the paragraphs above [2,3,4,6,7,8,9,10,11,12,13,14].

**Table 1:** Overview of the HF with Preserved Ejection Fraction Landmark Trials

TRIAL	POPULATION	INTERVENTION	RESULTS
CAMEO - DAPA	38 enrollees; duration of 24 weeks; mean patient age = 68; NYHA Class II-III LVEF³ 50%	Randomized to 10 mg dapagliflozin daily vs placebo	Reduced PCWP at 24 weeks vs placebo (at rest -6.6 vs -0.4 mmHg, P=0.027) (with exercise -2.5 vs + 1.1 mmHg (P=0.029)
CANVAS	Two multicenter, randomized, double-blind, placebo-controlled trials; N=10,142 (4330 in Canvas, 5812 in Canvas-R)	Randomized to canagliflozin 300 mg, canagliflozin 100 mg, or placebo daily	Reduction in CV mortality, nonfatal MI, or nonfatal stroke (26.9 vs 31.5 participants with an event per 1000 patient-years; HR 0.86; 95% CI, 0.75-0.97; P<0.001 for noninferiority=0.02 for superiority); Reduced hospitalizations for HF (5.5 vs 8.7 participants with an event per 1000 patient-years (HR 0.67; 95% CI 0.52-0.87)
CANVAS - R		Randomized in 1:1 ratio: canagliflozin at an initial dose of 100 mg daily with an optional increase to 300 mg weekly at week 13 vs placebo	
DAPA-HF	Multicenter, double-blind, parallel-group, randomized, controlled trial; N = 4744; Patients with HFrEF, EF£40% and NYHA II-IV; Ages ³ 18 years	Randomized to dapagliflozin 10 mg daily vs placebo	Reduced worsening HF (hospitalization or urgent care visit resulting in IV therapy for HF) or CV mortality (16.3% vs 21.2%; HR 0.74; 95% CI 0.65-0.85; P<0.001)
DECLARE - TIMI	Multicenter, double-blind, randomized controlled phase 3b trial; N=17,160; DM II; Age³ 40 years	Randomized to 10 mg dapagliflozin vs placebo daily	Reduced cardiovascular death or hospitalization for HF (4.9% vs 5.8%, CI 0.73-0.95, NNT=111)
EMPEROR - Preserve	Multicenter, double-blind, parallel-group, randomized, controlled trial; N=5988; NYHA Class II-IV LVEF >40% while clinically stable; Age ³ 18 years; BMI < 45 kg/m^2^	Randomized to empagliflozin 10 mg daily or placebo; Stratified by Geographic region, diabetes status, eGFR of 50, and LVEF 50%	Reduced death from cardiovascular causes or hospitalizations for HF (13.8% vs 17.1%; HR 0.79; 95% CI 0.69-0.90; P<0.001; NNT=30); Reduced hospitalizations for HF (8.6% vs 11.8%; HR 0.71; 95% CI 0.60-0.83; NNT=31)
EMPEROR – Reduced	Multicenter, multinational, double-blind, parallel-group, randomized, controlled trial; N=3730; NYHA II-IV; Age>18 years; HFrEF and NT-proBNP > 600pg/mL	Randomized to empagliflozin 10 mg daily or placebo in addition to usual therapy	Reduced composite of cardiovascular death and first hospitalization for decompensated HF (19.4% vs 24.7%; HR 0.75; 95% CI 0.65-0.86; P<0.001)
SOLOIST – WHF	Multicenter, phase 3, double-blind, randomized, placebo-controlled trial; N=1222	Randomized to 200 mg up to 400 mg sotagliflozin vs placebo	Reduced total CV death, HF hospitalizations, or urgent visits for HF (70 vs 98 per 100 patient-years; HR 0.67, 95% CI 0.52-0.85; P=0.0009); Reduced rate of primary endpoint per 100 patient-years (51.0 vs 76.3; HR 0.67, 95% CI 0.52-0.85, P<0.001)
SCORED	N=10584; DM II; CKD ±albuminuria	Randomized 1:1 to 400 mg sotagliflozin vs placebo daily	Reduced major adverse CV events defined as CV death, myocardial infarction, or stroke (8.4% vs 8.9%; HR 0.84, 95% CI 0.72-0.99, P=0.035

While there are many proposed mechanisms of how SGLT2 inhibitors are cardioprotective, it is not entirely clear. Table 2 summarizes some of the proposed suggested mechanisms [15,16,17,18,19,20].

**Table 2:** Proposed Cardioprotective Properties of SGLT2 Inhibitors

Proposed Cardioprotective Mechanisms of SGLT2 Inhibitors
1. Blood pressure lowering
2. Increasing diuresis/natriuresis
3. Improving cardiac energy metabolism
4. Preventing inflammation
5. Weight loss
6. Improving glucose control
7. Inhibiting the sympathetic nervous system
8. Preventing adverse cardiac remodeling
9. Preventing ischemia/reperfusion injury
10. Inhibiting the cardiac Na+/H+ exchanger
11. Inhibiting SGLT1
12. Reducing hyperuricemia
13. Increasing autophagy and lysosomal degradation
14. Decreasing epicardial fat mass
15. Increasing erythropoietin (EPO) levels
16. Increasing circulating provascular progenitor cells
17. Decreasing oxidative stress
18. Improving vascular function

Adverse effects related to SGLT 2 inhibitors

SGLT2 inhibitors, while offering promising therapeutic benefits in the management of diabetes and HF, are accompanied by several notable adverse effects. Among these, genital mycotic infections stand out as the most commonly reported adverse effect [21]. Additionally, urinary tract infections (UTIs) and pyelonephritis are associated with SGLT2 inhibitor use, possibly because of glucosuria, with dapagliflozin demonstrating a dose-dependent risk [22]. Another significant concern is the occurrence of diabetic ketoacidosis (DKA), which shows a threefold increase in risk with SGLT-2 inhibitors [23], including a higher incidence of euglycemic DKA [9], likely from non-insulin-dependent excretion of glucose. The risk of acute kidney injury (AKI) is also notable, likely stemming from volume depletion resulting from natriuresis [24]. Furthermore, the potential for hypoglycemia emerges, particularly when SGLT2 inhibitors are co-administered with insulin secretagogues and insulin [25]. These adverse effects emphasize the necessity for clinicians to carefully assess risks and benefits and implement appropriate monitoring strategies when prescribing SGLT2 inhibitors [26].

Discussion

Over the years, healthcare providers have used the same measures for HFrEF and HFpEF, with no proven advantage in patients who have HFpEF. Until recently, the use of MRAs and SGLT2 inhibitors was established as HFpEF-specific therapy. In the numerous trials we have mentioned for SGLT2 inhibitors, it is established that the use of such provides a mortality benefit.

There are numerous trials for the use of MRAs as well, such as the Treatment of Preserved Cardiac Function Heart Failure With an Aldosterone Antagonist (TOPCAT) trial, which showed that the use of MRA does indeed reduce hospitalization in patients with HFpEF, but it does not have an added benefit on mortality [27]. Additionally, larger-scale trials should be conducted to unlock the true potential of MRAs and their synergism when used together with SGLT2 inhibitors. 

The emergence of SGLT2 inhibitors as a promising therapeutic option in HF management has generated significant interest and investigation. While traditionally used as antidiabetic agents, SGLT2 inhibitors have demonstrated efficacy in slowing renal disease progression and reducing the risk of ESRD in both T2DM and HF patients, regardless of diabetes status. Notably, recent trials, such as EMPEROR-Preserved, have shown that SGLT2 inhibitors could potentially slow renal function decline and reduce HF-related hospitalizations in patients with HFpEF. 

Numerous clinical trials, including DAPA-HF, EMPEROR-Preserved, CANVAS Program, DECLARE-TIMI, SOLOIST-WHF, SCORED, and CAMEO-DAPA, have investigated the effects of various SGLT2 inhibitors on cardiovascular outcomes and mortality in HF patients. Overall, SGLT2 inhibitors have demonstrated significant reductions in cardiovascular adverse events, including cardiovascular death, hospitalizations for HF, and major adverse cardiovascular events, across different patient populations and risk profiles. These findings suggest a potential role for SGLT2 inhibitors in improving the outcomes and reducing morbidity in HF patients.

The proposed mechanisms underlying the cardioprotective effects of SGLT2 inhibitors are diverse and multifaceted, including blood pressure lowering, increasing diuresis, improving cardiac energy metabolism, preventing inflammation, weight loss, improving glucose control, and several others. These mechanisms collectively contribute to the potential benefits observed with SGLT2 inhibitor therapy in HF patients.

However, alongside the therapeutic benefits, SGLT2 inhibitors are associated with several notable adverse effects. These include genital mycotic infections, UTI, pyelonephritis, DKA, AKI, and the potential for hypoglycemia. These adverse effects highlight the importance of careful patient selection, monitoring, and risk-benefit assessment when prescribing SGLT2 inhibitors in clinical practice.

## Conclusions

SGLT2 inhibitors represent a promising therapeutic option in the management of HF, offering potential benefits in reducing cardiovascular events and improving outcomes. However, further research is needed to fully understand their effects on mortality and better understand the mechanisms underlying their cardioprotective effects. Additionally, ongoing surveillance for adverse effects and careful patient management are essential to optimize the use of SGLT2 inhibitors in HF patients.
